# The Neuroimmunome of Hepatitis Patients Associates With Disease Severity

**DOI:** 10.1002/jmv.70742

**Published:** 2025-12-05

**Authors:** Adriel Leal Nóbile, Anny Silva Adri, Júlia Nakanishi Usuda, Fernando Yuri Nery do Vale, Yohan Lucas Gonçalves Corrêa, Débora Gomes de Albuquerque Freitas, Roseane Galdioli Nava, Pedro Marçal Barcelos, Lena F. Schimke, Taj Ali Khan, Renato Santana de Aguiar, Niels Olsen Camara, Gustavo Cabral‐Miranda, Rodrigo J. S. Dalmolin, Helder I. Nakaya, Luiz Fernando Onuchic, Haroldo Dutra Dias, Igor Salerno Filgueiras, Otavio Cabral‐Marques

**Affiliations:** ^1^ Laboratory of Psychoneuroimmunology, Selye Lab University of São Paulo School of Medicine São Paulo Brazil; ^2^ Department of Clinical and Toxicological Analyses, School of Pharmaceutical Sciences University of São Paulo (USP) São Paulo SP Brazil; ^3^ Interunit Postgraduate Program on Bioinformatics, Institute of Mathematics and Statistics (IME) University of Sao Paulo (USP) São Paulo SP Brazil; ^4^ Department of Medicine, Division of Molecular Medicine, Laboratory of Medical Investigation 29 University of São Paulo (USP) School of Medicine São Paulo SP Brazil; ^5^ Public Health Reference Laboratory Khyber Medical University Peshawar Pakistan; ^6^ Department of Genetics, Ecology and Evolution, Institute of Biological Sciences Universidade Federal de Minas Gerais Belo Horizonte MG Brazil; ^7^ Department of Immunology, Institute of Biomedical Sciences University of São Paulo São Paulo SP Brazil; ^8^ Bioinformatics Multidisciplinary Environment Federal University of Rio Grande do Norte Brazil; ^9^ Hospital Israelita Albert Einstein São Paulo Brazil; ^10^ DO'R Institute for Research São Paulo Brazil

**Keywords:** Hepatocellular Carcinoma, Neuroimmune, Viral Hepatitis

## Abstract

Hepatitis is a systemic disease marked by neuroimmune dysregulation beyond hepatic inflammation. Using a systems biology approach, we conducted transcriptomic meta‐analyses across in vitro models, liver tissues, and PBMCs from hepatitis virus‐infected patients to identify neuroimmune signatures. We found a robust neuroimmunome signature, with neuroimmune‐related genes showing consistent differential expression across datasets. Functional enrichment revealed disruptions in neurotransmission (including synaptic, glutamatergic, noradrenergic and neuregulin pathways) and immune signaling (such as cytokines, interleukin‐1 response, T cell receptor, and trans‐synaptic signaling). Linear discriminant analysis (LDA) demonstrated that neuroimmune genes can predict disease severity. Several of these genes were also altered in hepatocellular carcinoma (HCC) samples from The Cancer Genome Atlas Program (TCGA), implicating them in oncogenic transformation. Ligand‐receptor analysis revealed dysregulated neuroimmune interactions in liver tissue, notably involving DBH‐ADRA1A/B/D, ADRA2A/B/C, ADRB1/2/3, IL33‐IL1RL1, and NRG1‐ERBB4. Critically, we observed an inverse correlation between neuroimmune gene expression and inflammation markers in advanced HCC, suggesting that neuroimmune suppression may facilitate immune evasion. These findings highlight the neuroimmunome as a potential biomarker and therapeutic target in hepatitis and its complications, reinforcing the role of neuroimmune crosstalk in liver disease progression.

## 1 Introduction

Hepatitis is a systemic disease characterized by liver inflammation, which can be caused by hepatitis viruses (HAV, HBV, HCV, HDV, and HEV), alcohol‐related liver damage, autoimmune hepatitis, or even excessive cigarette use [1, 2, 3, 4]. In severe cases, it can progress to hepatocellular carcinoma (HCC), a condition often associated with delayed diagnosis and the appearance of extrahepatic symptoms [5]. According to the World Health Organization (WHO), the number of lives lost to viral hepatitis is increasing, making it the second leading infectious cause of death globally, with approximately 1.3 million deaths each year [6, 7].

Despite their distinct genomic structures and clinical trajectories, including differences in transmission, potential for chronicity, and risk for extrahepatic manifestations, the five hepatitis viruses target the liver as their primary site of infection, triggering inflammatory and immune responses that impact systemic physiology [8, 9]. While HAV and HEV are generally associated with acute, self‐limiting infections, they serve as comparative biological references to contrast against the chronic inflammatory and oncogenic contexts of HBV, HCV, and HDV, which are well known to progress toward HCC and exhibit extrahepatic neurological and psychiatric manifestations [5, 10].

Additionally, although all five viruses have been shown to infect or interact with peripheral blood mononuclear cells (PBMCs) to varying extents, their ability to induce systemic immune alterations differs. This diversity provides a robust framework to investigate how chronicity, tissue tropism, and immune activation levels contribute to neuroimmune signatures [11, 12]. Due to the limited availability of acute‐phase PBMC and liver datasets for HAV and HEV, their inclusion in our analyses was primarily for comparative purposes, while the core meta‐signature was derived from HBV, HCV, and HDV, which are more directly implicated in HCC and sustained neuroimmune dysregulation [13, 14, 15].

The neuroimmune interface includes a range of soluble mediators, such as cytokines and neurotransmitters, which can impact neuronal activity and synaptic modulation [16, 17]. Notably, leukocytes have been observed to synthesize and release neurotransmitters, indicating that these immune cells are not merely passive responders but may actively participate in modulating neuronal signaling [18]. This crosstalk suggests that leukocytes form ‘immunological synapses’, microglia actively remodel synapses via complement‐dependent elimination, reinforcing the idea that immune cells are both receptive to and embedded within neuronal signaling networks [16, 19].

Additionally, a chronically inflamed liver becomes a fertile ground for neuroimmunological dysregulation. From tumor initiation to metastasis, cancer cells in the liver can secrete cytokines and chemokines that modulate both the immune and nervous systems [20]. Conversely, the immune microenvironment significantly influences tumor progression, and neural signals, mediated through neurotransmitters, critically affect tumor behavior [21, 22]. For example, autonomic nerve fibers promote blood vessel proliferation, supporting tumor growth, and tumors can activate brainstem neurons, which suppress the activity of immune cells such as PBMCs [23].

To investigate this, we performed an integrative transcriptomic analysis combining in vitro infection models, liver biopsy samples from HCC patients with seropositive hepatitis versus non‐tumoral regions, and PBMCs from individuals positive for hepatitis virus compared to seronegative controls. Our study aimed to uncover virus‐specific and shared molecular signatures, particularly focusing on the neuroimmunome, defined as a set of genes that mediate the interface between the nervous and immune systems.

## 2 Materials and Methods

We employed a systems biology framework to explore the neuroimmune transcriptomic landscape in hepatitis virus infections and HCC progression, as detailed in Figure S1.

All analyses were conducted with R in RStudio (v. 4.3.2), using Bioconductor and CRAN packages, with full reproducibility ensured via workflows and R scripts available on GitHub: https://github.com/adrielleal/neuroimmunome_hcc_severity. The data and tables used in our analyses are available as supporting files, including input datasets, variables and parameters, as well as processed results and statistical metrics supporting our findings, also available on GitHub.

Details of data curation, metadata structure, viral case confirmation, including identification of chronic and acute phases of viral hepatitis, the diagnostic methodologies used, differential expression and meta‐analyses, as well as enrichment analyses and machine learning models, are provided in Supporting information 1.

### 2.1 Diseasome Analysis

To assess whether the genes associated with tumor grade were specifically related to HCC, we performed a disease enrichment analysis using DisGeNET via the EnrichR platform [24]. This approach integrates data on gene‐disease associations across a wide spectrum of genetic conditions, disorders, and syndromes, drawing from scientific literature and genetic variant databases.

### 2.2 Ligand–Receptor Analysis

CellChat was used as a reference to annotate known ligand–receptor interactions among selected genes, including meta‐DEGs, synaptic markers, and HCC‐associated transcripts. This enabled the identification of signaling axes potentially involved in neurotransmission and immune modulation [25].

## 3 Results

### 3.1 A Conserved Neuroimmunological Signature Across *in Vivo* Hepatitis Viruses

**Figure 1:**
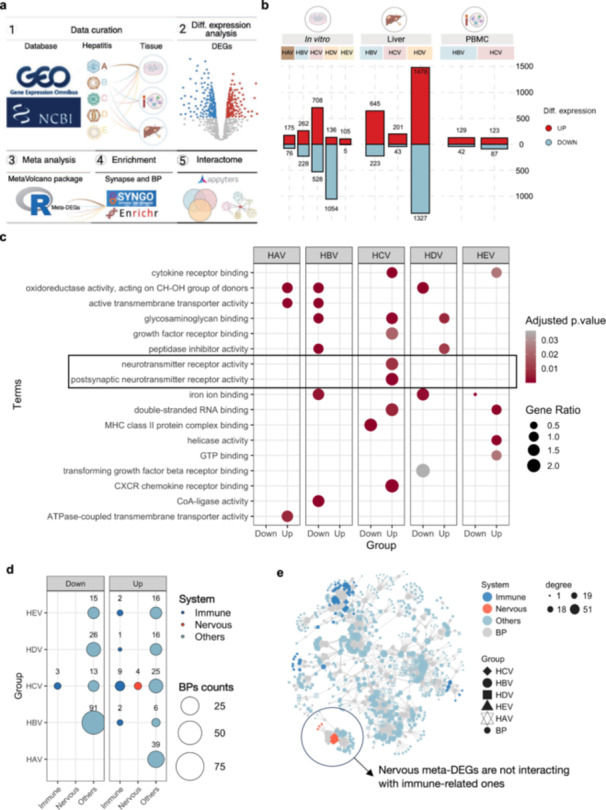
A conserved neuroimmune signature across hepatitis virus infections. (a) Schematic overview of the analytical workflow. Public transcriptomic datasets from in vitro models, liver tissues, and PBMCs infected with hepatitis A–E viruses (HAV–HEV) were curated from GEO/NCBI and analyzed using *R*‐based pipelines for differential expression (DESeq. 2/Limma), meta‐analysis (MetaVolcanoR), and functional enrichment (EnrichR, SynGO, Appyters). (b) Bar plot summarizing the number of meta‐significantly upregulated (red) and downregulated (blue) genes across virus types and sample sources. HDV‐infected liver tissue displayed the highest degree of transcriptional dysregulation. (c) Dot plot of enriched BPs among upregulated and downregulated genes for each hepatitis virus (HAV–HEV). Notably, neurotransmitter receptor activity and postsynaptic receptor activity were enriched in HBV, HCV, and HDV samples. Dot size represents the gene ratio; color intensity reflects statistical significance (FDR‐adjusted *p*‐value). (d) Proportion of enriched terms categorized by biological system (immune, nervous, or other) across viruses and regulation direction (up/down). HBV and HCV showed the highest enrichment in immune and nervous system–related functions. (e) Network visualization of enriched biological processes and functional terms, colored by system classification. Node size indicates connectivity degree, and node shape reflects the viral group in which the enrichment occurred. A distinct neuroimmune module involving synaptic signaling emerged predominantly in HCV and HBV networks.

Neuroimmune interactions are increasingly recognized as key contributors to the pathophysiology of chronic liver diseases, including their progression toward HCC and the manifestation of extra‐hepatic symptoms [21, 22, 26]. To better understand this interface, we performed a comprehensive transcriptomic analysis across multiple experimental contexts (Figure 1a,b; Figure S3). Despite the association of the nervous system with HCV (Figure 1c), functional enrichment analysis in in vitro hepatic models (PHH, HepG2, Huh7) revealed an absence of a neuroimmune signature (Figure 1d,e). These findings highlight the critical role of complex tissue microenvironments in facilitating inter‐system crosstalk and caution against overreliance on in vitro data for neuroimmune inferences drawn from PBMC and liver tissue analyses [27, 28, 29].

**Figure 2:**
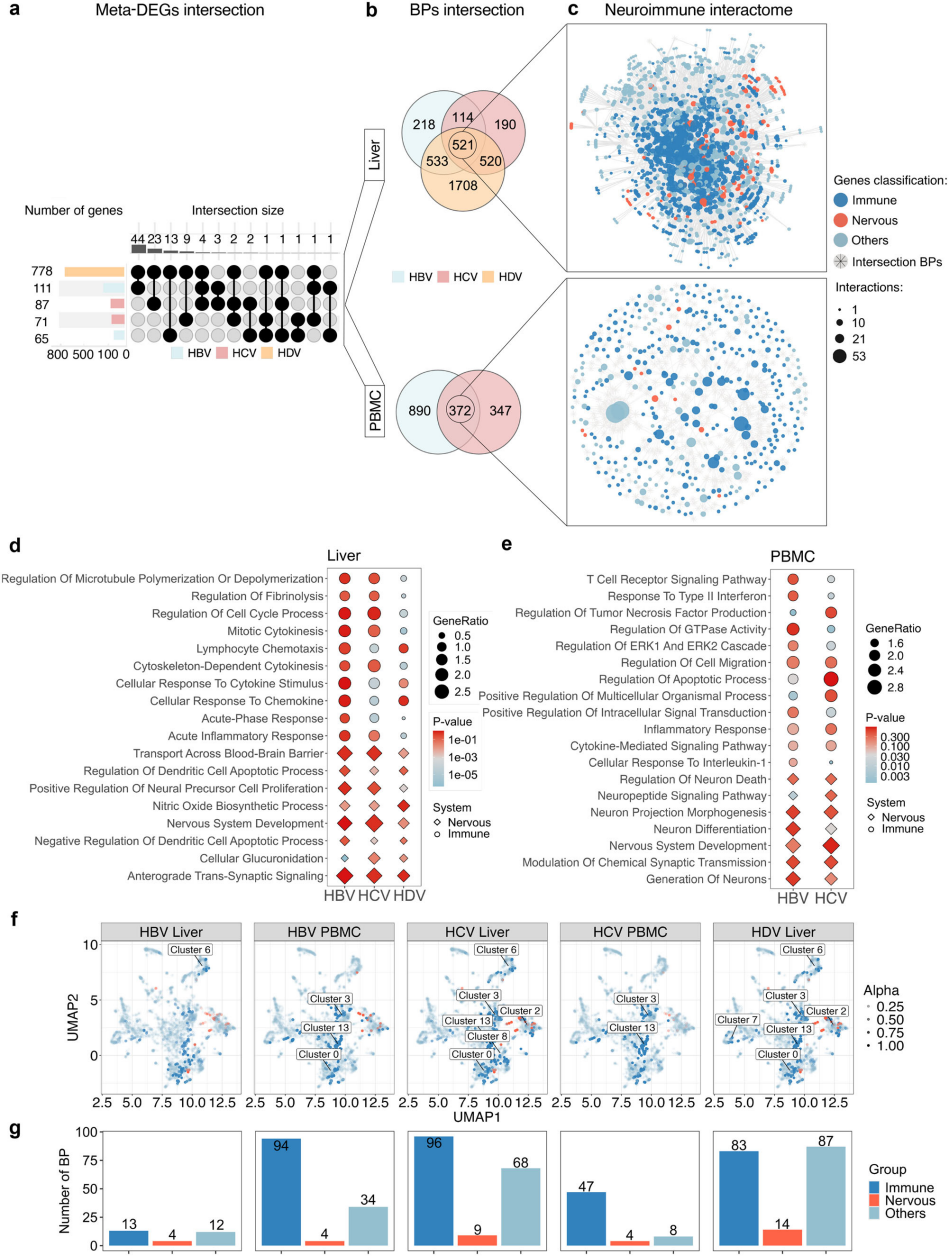
Enrichment of receptor activity and neuroimmune network topology across hepatitis virus infections in in vitro conditions. (a) Upset plot showing limited overlap of meta‐DEGs across liver and PBMC conditions, indicating context‐specific transcriptional responses. (b) Functional enrichment of viral hepatitis meta‐DEGs revealed intersecting BPs, despite limited gene‐level intersection. (c) Based on keyword annotations, genes were categorized as nervous system–related (orange), immune‐related (blue), or others (light blue), with shared BPs depicted in gray. Node size and configuration represent the number of interactions. (d) Top 20 most significantly enriched biological processes (based on *p*‐value) that were common among the oncogenic hepatitis viruses, HBV, HCV, and HDV, in liver tissue, and (e) in PBMCs. In both figures, the shape of the dots represents the keyword category: diamonds for nervous system–related terms and circles for immune‐related terms. (f) UMAP projection of enriched biological processes (BPs), clustered using TF‐IDF and Leiden algorithms. Each point represents a GO term, positioned according to gene set similarity. Clusters containing ≥ 10 significantly enriched terms (FDR < 0.05) are labeled. Point transparency (alpha) indicates the density of significant terms per cluster, with darker points reflecting higher counts, clusters 2 and 0 presents the most nervous classified BPs. (g) Bar plots summarizing the number of enriched BPs categorized as nervous system‐related (orange), immune‐related (blue), or other (light blue) across virus–tissue conditions. HDV liver samples showed the highest number of enriched terms from both functional domains.

Despite limited intersection in meta‐DEGs across virus–tissue combinations (Figure 2a), functional enrichment revealed a convergent neuroimmune signature in both liver and PBMC datasets, with a total of 521 enriched BPs in the liver and 372 in PBMCs shared among viral hepatitis enrichment meta‐DEGs (Figure 2b,c); Figures S4a,b and S5a). These included cytokine‐mediated signaling, interleukin‐1 response, T cell receptor signaling, anterograde trans‐synaptic signaling, and synaptic transmission modulation. We highlight the top 20 biological processes (10 immune‐related and 10 nervous‐related) in which meta‐DEGs were commonly enriched and statistically significant (adjusted *p*‐value < 0.05) among viral hepatitis (HBV, HCV, HDV) in liver (Figure 2d) and PBMC (Figure 2e) tissues.

Despite transcriptional heterogeneity driven by host tissue, viral structure, and tropism, enriched BPs across viruses revealed shared functional programs. Among these, HDV infections exhibited the broadest diversity of immune‐ and nervous‐related enrichments, consistent with its known clinical severity [1, 30].

Additionally, dimensionality reduction and clustering of enriched BPs positioned neuroimmune‐related terms in close proximity (Figure 2f,g), suggesting partial convergence of nervous and immune system processes within shared transcriptional programs. While no discrete neuroimmune modules were defined, the spatial distribution highlights recurring functional themes across viruses and tissues that may reflect integrated, cross‐system responses.

### 3.2 Neuroimmune Genes Stratify Hepatitis‐Infected Tissues

**Figure 3:**
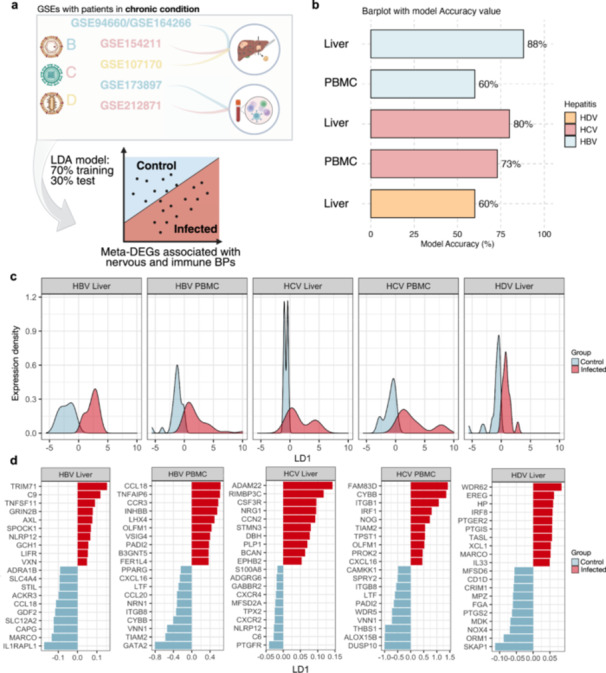
Neuroimmune identity codes distinguish infected tissues from healthy states. (a) Linear discriminant analysis (LDA) pipeline applied to five GEO datasets containing nervous and immune‐related meta‐DEGs. Samples were split into training (70%) and test (30%) sets to model infection stratification in liver and PBMC tissues. (b) Bar plot showing LDA model classification accuracy across datasets. Most models performed well in distinguishing infected from control samples, particularly in liver‐derived data. (c) Density plots of the first linear discriminant component (LD1) from LDA models across each data set. Red and blue curves represent infected and control groups, respectively, illustrating strong class separation along the x‐axis, which represents LD1 discriminant scores. The y‐axis denotes the expression density of sample distributions. (d) Top 10 genes ranked by linear discriminant analysis (LDA) for sample stratification between infected and control groups. Bar plots display LDA coefficients (LD1) along the x‐axis, with red bars indicating genes contributing most to the separation of infected samples, and blue bars indicating genes associated with controls. Each panel represents a distinct virus–tissue combination.

To further evaluate the stratification capacity of neuroimmunome‐associated meta‐DEGs in distinguishing viral hepatitis infections from healthy states, we applied a linear discriminant analysis (LDA) model across PBMCs from patients and healthy controls, as well as between infected and noninfected liver tissue samples (Figure 3a,b). The analysis demonstrated that neuroimmunome‐associated meta‐DEGs robustly discriminated HBV, HCV, and HDV infection states from noninfected controls (Figure 3c).

The LDA revealed that several genes linked to immune activation, inflammation, and cellular stress responses, including *TRIM71*, *CCL18*, *ADAM22*, *FAM83D*, *and WDR62*, emerged as major contributors to the classification. These immune‐related signatures underscored the systemic activation of inflammatory pathways across different viral infections.

Importantly, nervous system‐associated genes, particularly those related to synaptic function and neurotransmission, also contributed significantly to group separation. Genes involved in presynaptic organization, synaptic signaling, and neurotransmitter regulation, such as *GRIN2B*, *NRG1*, *OLFM1*, and *CAMKK1*, were among the top discriminators. This finding reinforces the concept that synaptic‐associated molecular programs, traditionally linked to the nervous system, are also embedded within the immune system and actively contribute to immune regulation during viral infections. Together, these results suggest the presence of neuroimmunological gene signatures that span both systemic and tissue‐specific immune compartments. However, while coordinated regulatory patterns were observed, the contribution of conserved versus virus‐specific genes remains to be fully resolved, as key discriminative genes were not consistently shared across all conditions.

Ligand‐receptor interactions derived from LDA‐selected genes revealed that neurotransmitter signaling axes were predominantly enriched in liver tissues, with no corresponding circuits detected in PBMCs. Noradrenergic signaling emerged as a shared module across HBV (blue), HCV (red), and HDV (orange) liver samples, involving transporters (e.g., SLC18A1/2, SLC6A2) and receptors from the ADRA and ADRB families. HDV liver samples uniquely exhibited EGF–EPHB signaling, while NRG1–ERBB interactions were restricted to HCV and HDV livers. Additionally, glutamatergic components (SLC17A6–A8, GRIN1, GRIN2B) were linked to HBV and HCV infections. The integrated signaling landscape converged on receptor families including ADRA, ADRB, ERBB, GRIN, and EPHB, supporting the existence of virus‐specific yet overlapping neuroimmune communication modules in hepatic environments (Figure S5).

### 3.3 Exploring the Association of Meta‐DEGs With Synapse Ontologies

**Figure 4:**
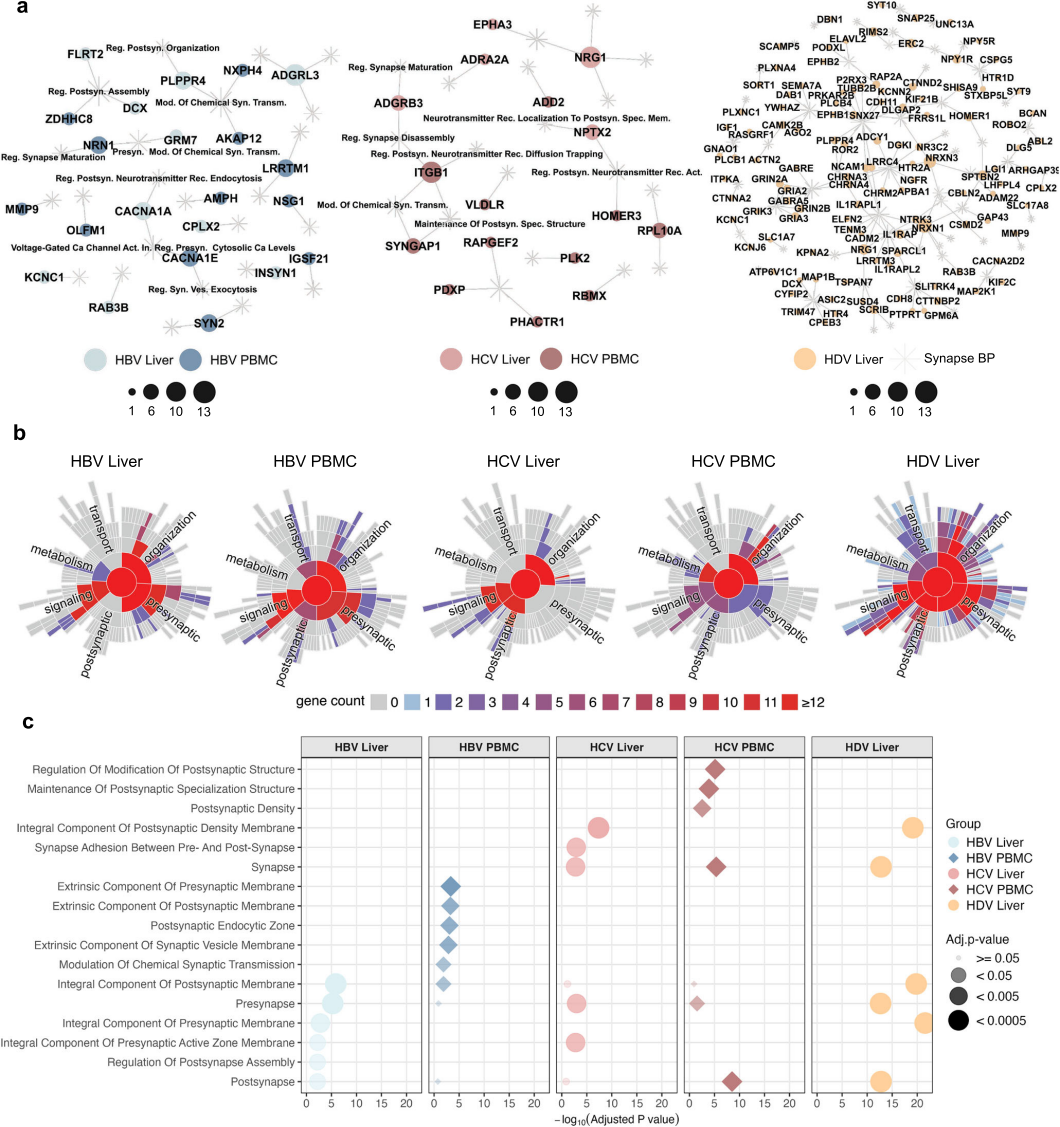
Synaptic features emerge within the neuroimmune transcriptome across hepatitis infections. (a) Network representations of synaptic‐related meta‐DEGs enriched for synaptic ontologies in liver and PBMC samples, stratified by HBV (left), HCV (middle), and HDV (right). The networks highlight the most significant Biological Processes (BP), cellular components (CC), and genes with multiple direct functional interactions. Nodes represent genes; edges indicate known functional relationships. (b) Circular dendrograms displaying enriched Gene Ontology (GO) terms related to synaptic biology for each condition (HBV liver, HBV PBMC, HCV liver, HCV PBMC, HDV liver). Segments illustrate functional diversity, including terms associated with vesicle transport, membrane dynamics, and chemical synaptic signaling. (c) Dot plot summarizing the enrichment of postsynaptic, presynaptic, and synaptic membrane‐related GO terms across virus–tissue groups. Dot size represents statistical significance (FDR‐adjusted *p*‐value); the x‐axis shows the –log₁₀(Adjusted *P* value) based on enriched genes. Color and shape denote virus and tissue types, respectively. Enrichment of synaptic structures was observed in both hepatic and circulating immune compartments, particularly under HDV and HCV infections.

There is a growing recognition that leukocytes synthesize and release neurotransmitters, which bind to receptors on immune and nonneural cells [17, 22], suggesting the presence of synaptic‐related proteins. To investigate this in the context of viral hepatitis, we analyzed whether meta‐DEGs identified in hepatic tissues and PBMCs were enriched for synapse‐related BPs. This analysis confirmed that genes formed an interaction network (Figure 4a) with significant associations with processes such as presynaptic organization, synaptic signaling, and neurotransmitter regulation (Figure 4b,c).

Furthermore, by exploring ligand–receptor interactions across virus–tissue contexts, we identified distinct neurotransmission modules in HBV, HCV, and HDV liver samples, involving glutamatergic (GRIN2B, SLC1A), noradrenergic (DBH, SLC6A2), and neuregulin (NRG1, ERBB) pathways. Receptors such as ADRA1A–C and ERBB4 exhibited virus‐specific connectivity, suggesting context‐dependent engagement of synaptic signaling (Figure. S6a). Functional enrichment of these ligand–receptor meta‐DEGs reinforced their association with synaptic transmission, glutamate signaling, and axon guidance. Enriched cellular components included postsynaptic membranes, neuromuscular junctions, and NMDA receptor complexes (Figure. S6b).

Hence, our results reinforce the emerging view that elements of synaptic biology are not confined to the nervous system but are also embedded within immune cells, potentially shaping their regulatory behavior in both systemic and tissue‐specific immune responses [21, 31, 32].

### 3.4 Sustained Neuroimmunome Dysregulation Across Viral Hepatitis and HCC Severity

**Figure 5:**
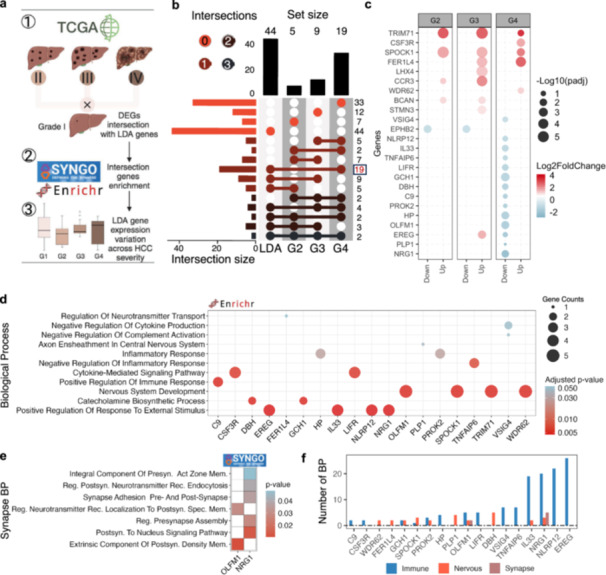
Neuroimmune segregated genes bridge hepatitis infection to hepatocellular carcinoma severity. (a) Schematic overview illustrating the integration of viral hepatitis‐derived LDA signatures with hepatocellular carcinoma (HCC) severity, stratified by TCGA grades. Neuroimmune genes identified in infection contexts were intersected with differentially expressed genes across HCC stages (G1–G4) using TCGA‐LIHC data. (b) UpSet plot illustrating the overlap between LDA‐derived infection‐associated genes and differentially expressed genes (DEGs) across HCC histological grades. A core set of 19 intersecting genes was identified, most of which were progressively upregulated in advanced tumors (G4) compared to low‐grade cases (G1). (c) Dot plot of expression changes (log2FC) for the 19 intersecting genes across HCC grades G2–G4, highlighting the consistent upregulation of immune–neural genes in late‐stage tumors. (d) Functional enrichment of the intersecting gene set using EnrichR. Terms include immune signaling, neurotransmitter transport, and synaptic development, suggesting dual roles in immune and nervous system regulation. (e) SynGO‐based enrichment analysis of synapse‐related genes. Processes include presynaptic membrane organization, neurotransmitter receptor localization, and synaptic adhesion involving *OLFM1* and *NRG1* genes. (f) Bar plot showing the number of BPs associated with each of the 19 intersecting genes across three functional categories: immune (blue), nervous (red), and synaptic (brown). Most genes were predominantly linked to immune‐related processes; however, several, including *NRG1*, *IL33*, *OLFM1*, and *DBH*, were also enriched for nervous system and synaptic functions, highlighting their dual regulatory roles in infection and tumor progression.

Given the progressive nature of viral hepatitis and its role as a major risk factor for HCC, we investigated whether infection‐associated transcriptional alterations extend along the HCC progression axis (Figure 5a). To this end, we assessed the overlap between meta‐DEGs previously ranked by LDA as discriminative for infected samples and tumor‐related DEGs across different tumor grades.

We observed a progressive increase in this overlap, with the strongest convergence occurring in advanced tumors. Specifically, 19 genes overlapped between LDA‐ranked meta‐DEGs and those identified as differentially expressed in grade 4 versus grade 1 tumors (Figure 5b). Therefore, in a broader analysis, we identified 24 genes that overlapped with LDA results and exhibited progressively divergent expression patterns across tumor grades, based on differential expression comparisons between G4, G3, and G2 versus G1. Several of these genes were consistently up‐ or downregulated in high‐grade tumors (Figure 5c).

Focusing on the most severe tumor grade, functional enrichment analysis of the 19 genes shared between LDA‐ranked meta‐DEGs and G4 DEGs revealed a dual role, with significant enrichment in both immune‐related and nervous system‐related BPs (Figures 5d, and S7a–d). Of particular interest, synapse‐associated pathways such as neurotransmitter receptor endocytosis and synaptic adhesion complexes were enriched, implicating key regulators such as *OLFM1* and *NRG1* (Figure 5e,f). These findings suggest that neuroimmunological remodeling is not only triggered during viral infection but is also sustained and amplified during tumor progression.

Notably, several of the identified genes exhibited pleiotropic functions, integrating immune and neural signaling axes. For instance, *C9* is related to cytokine production, complement activation, and synaptic pruning, bridging innate immune responses and nervous system remodeling. *CSF3R* is involved in cytokine‐mediated immune regulation and myeloid cell activation. Similarly, *NRG1* and *PLP1* play roles in nervous system development and axon ensheathment, indicating persistent engagement of neurodevelopmental programs. *DBH* is associated with catecholamine biosynthesis, consistent with its function in norepinephrine production and neuroimmune modulation. Inflammatory mediators such as *TNFAIP6* integrate inflammasome activation, and *NLRP12* facilitates extracellular matrix remodeling, while *FER1L4*, *HP*, and *TRIM17* were predominantly enriched in classical immune pathways (Figure 5f).

Together, these results uncover a complex interplay between synaptic structure, neurotransmission, and immune cell activity in the context of viral infection and tumor progression. This suggests that neuroimmunome dysregulation constitutes a persistent molecular signature from infection through oncogenic transformation [21, 26].

### 3.5 Transcriptomic Shifts Reflect the Decoupling of Neuroimmune Homeostasis in Late‐Stage Disease

**Figure 6:**
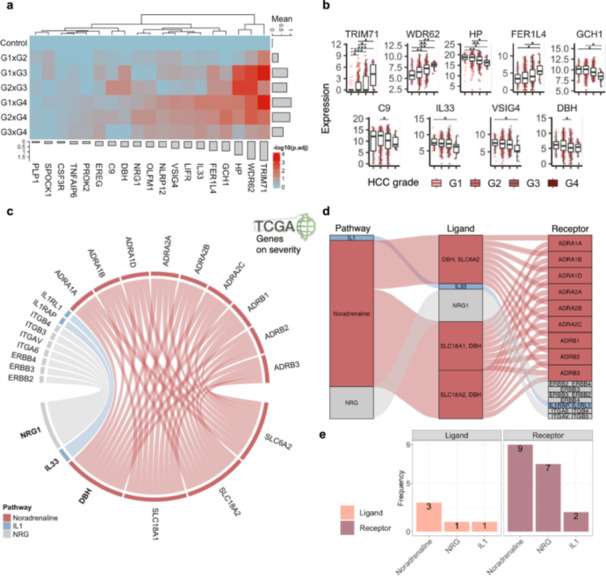
Noradrenergic and synaptic signaling patterns throughout HCC progression. (a) Heatmaps show hierarchical clustering of the 19 infection‐associated genes across HCC severity, with Wilcoxon comparisons (e.g., G1 vs. G2, G1 vs. G4). The color scale reflects expression –log10(*p*. adj value). The progressive upregulation of neuroimmune genes is most pronounced in G3–G4 tumors. (b) Boxplots of representative neuroimmune genes exhibit significantly altered expression patterns across tumor grades (G1–G4), including TRIM71, WDR62, HP, FER1L4, GCH1, IL33, VSIG4, and DBH. (c) A chord diagram ofillustrates ligand–receptor interactions derived from variedvarious genes, stratified by neuroimmune signaling axes. Noradrenaline (DBH, SLC6A2), neuregulin (NRG1), and IL‐1 (IL33) pathways exhibitshow extensive associations with adrenergic (ADRA, ADRB), ERBB, and immune‐related receptors. (d) A Sankey diagram details ligand‐receptor pairings for noradrenergic, IL‐1, and NRG1 signaling. Arrows trace connections from source genes (left) to multiple receptors (right), with DBH and NRG1 serving as key interaction hubs. (e) Frequency plots summarize the number of ligands and receptors associated with each signaling pathway. Noradrenaline and NRG1 circuits dominate receptor interactions, highlighting their expanding role in late‐stage HCC.

**Figure 7:**
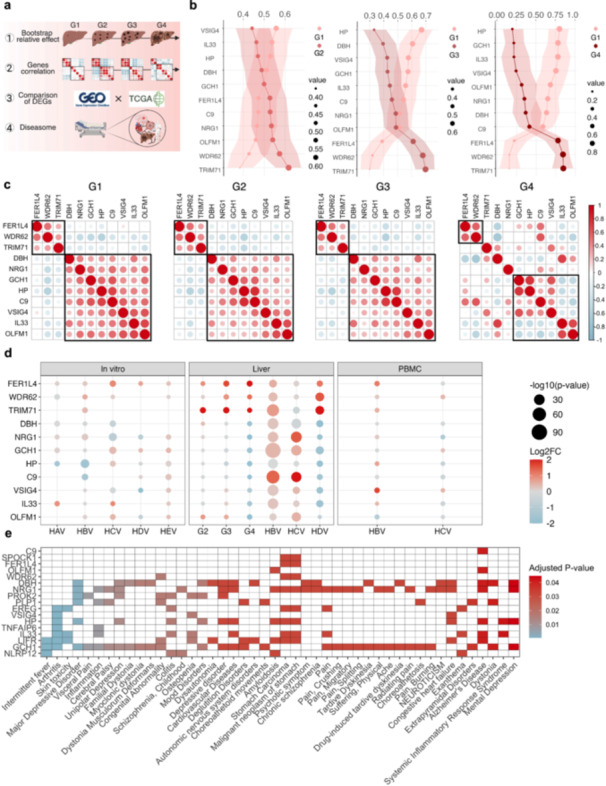
Breakdown of neuroimmune coordination accompanying tumor progression and extrahepatic dysfunction. (a) An overview of the analytical workflow used to investigate neuroimmune gene dynamics across HCC stages. Steps include bootstrap‐based relative effect analysis size comparisons across grades; genegene correlation analysis; DEG comparisons between infection and HCC datasets; and disease enrichment analysis via DisGeNET. (b) Relative effects, show the progressive divergence in expression levels (mean values) for selected neuroimmune and synaptic genes (*OLFM1* and *NRG1*) across early (G1) and late (G2‐G4) HCC grades. Shaded areas indicate confidence intervals for each group comparison. (c) Correlation matrices of neuroimmune genes for HCC grades G1 to G4, based on Spearman correlation coefficients. Early‐stage tumors (G1–G2) display coherent co‐expression networks that are progressively disrupted in G3‐G4, reflecting the decoupling of neuroimmune coordination. (d) Dot plots show the expression of key neuroimmune meta‐DEGs across viral infections (HAV‐HEV), HCC grades, and tissue types. Several genes, including *FER1L4, DBH, WDR62, and* NRG1, exhibit consistent deregulation across both infection and tumor contexts, supporting their role in systemic dysregulation. (e) A heatmap of disease enrichment analysis using DisGeNET reveals that several neuroimmune genes associated with HCC progression in our analysis also mapped to a broad spectrum of other diseases, including neuropsychiatric, autoimmune, and inflammatory disorders (e.g., major depressive disorder, schizophrenia, arthritis, and systemic lupus). These findings reinforce the relevance of these genes in extrahepatic pathology and diverse clinical contexts.

Building on the observed convergence between viral infection and tumor progression, we further evaluated the transcriptional dynamics of the 19 genes that overlapped between LDA viral hepatitis results and HCC severity on G4. Our statistical analyses reveal significant grade‐dependent changes in the expression of these relevant genes for HCC progression, particularly among key neuroimmune regulators (Figure 7e). Hierarchical clustering of adjusted *p*‐values from Wilcoxon statistical analysis results revealed a progressive modulation of these genes across HCC grades, with marked shifts in expression profiles particularly evident between early (G1xG2) and advanced (G3xG4) stages (Figure 6a).

Specifically, genes such as *TRIM71*, *DBH*, *HP*, *FER1L4*, and *GCH1* exhibited dynamic and progressive expression changes along tumor grades, with significant increases in Grade 4 tumors compared to Grade 1 (Wilcoxon test, adj. *p* < 0.05) (Figure 6b). These expression trends reinforce that neuroimmune signaling components are not only disrupted during viral infection but also become increasingly deregulated as tumors evolve toward higher malignancy.

To further explore how these nine genes relevant to HCC grading (*TRIM71*, *WDR62*, *HP*, *FER1L4*, *GCH1*, *C9*, *IL33*, *VSIG4 and DBH*) might be involved in the neuroimmunome, we performed a neurotransmitter ligand‐receptor interaction analysis. The analysis revealed three major neuroimmune signaling axes anchored around noradrenaline, IL‐1, and NRG pathways (Figure 6c). Among these, noradrenaline signaling emerged as the most prominent, characterized by the differential expression of key ligands such as DBH and subunits, SLC6A2, and coordinated overexpression of their receptors across the ADRA and ADRB families. Additionally, IL‐1 and NRG pathways exhibited biological connectivity, with *IL33* and *NRG1* acting as shared ligands linking to multiple receptors (Figure 6d,e).

Together, these findings suggest the existence of a sustained neuroimmune signaling framework along HCC progression, with potential involvement of noradrenergic, NRG, and IL pathways. While the current ligand‐receptor analysis indicates a comparable contribution of each axis, they may represent a link between chronic inflammation, synaptic‐like immune modulation, and malignant transformations [17, 21, 26, 31, 32].

### 3.6 Progressive Breakdown of Neuroimmune Synchrony Across Hepatocellular Carcinoma Grades

To further characterize the neuroimmune transcriptional landscape during HCC progression, we analyzed the expression dynamics of nine neuroimmune‐associated genes that exhibited significant differential expression across cancer grades (G1xG2, G1xG3, G1xG4), along with synapse‐related genes, *OLFM1* and *NRG1*, identified as differentially expressed between Grade 1 (G1) and Grade 4 (G4) tumors (Figure 7a). Analysis of TCGA HCC cohorts revealed a progressive decline in the expression of these genes with increasing disease severity, with the most pronounced reductions observed in Grade 4 tumors. Using relative effects analysis with non‐parametric MANOVA bootstrap resampling, we confirmed that these transcriptional shifts were statistically significant, particularly distinguishing early‐stage (G1–G2) from late‐stage (G3–G4) tumors (Figure 7b).

Importantly, this transcriptional decline was accompanied by a collapse of gene‐gene correlation networks. In early‐stage tumors (G1–G2), coherent positive correlations were observed among key neuroimmune and synapse‐associated transcripts, including *IL33*, *OLFM1*, *FER1L4*, and *NRG1*, forming integrated neuroimmune modules (Figure 7c). However, these coordinated relationships became attenuated or inverted in advanced stages. Notably, *IL33* and *OLFM1*, initially positively correlated with immune‐related genes, became negatively associated in G4 tumors, reflecting a breakdown in neuroimmune synchrony and signaling integration.

Further comparison analysis revealed that deregulation of *FER1L4*, *DBH*, and *NRG1* was consistently observed across liver tissue and PBMCs in patients with chronic viral hepatitis infections (HBV, HCV, and HDV), suggesting that the observed disturbances are not purely tumor‐intrinsic but may reflect systemic immune and metabolic stress associated with chronic infection (Figure 7d).

Gene and disease association analyses further revealed that several key neuroimmune transcripts, including *DBH*, *FER1L4*, *WDR62*, and *NRG1*, were significantly linked to a spectrum of pathological conditions spanning neuropsychiatric disorders (e.g., major depressive disorder), autoimmune diseases, and cancer‐related syndromes (Figure 7e). These findings highlight the conserved involvement of neuroimmune regulators in diverse pathological processes and position the liver as a critical hub within broader systemic disease networks.

## 4 Discussion

This study reveals a conserved and adaptable neuroimmune landscape that spans viral hepatitis, systemic inflammation, and HCC. Marked by the expression of synaptic‐associated genes in both hepatic tissue and circulating immune cells, this molecular program challenges traditional boundaries between neural and immune systems. As HCC progresses, the disruption of neuroimmune synchrony and the emergence of neurotransmitter‐driven signaling, particularly via the noradrenergic axis, highlight a broader physiological integration between neural signaling and immune regulation. The liver emerges as a central hub in a multiorgan network, revealing targets at the metabolism‐immunity‐cancer interface. Cross‐tissue infection transcriptomics uncovered a systemic neuroimmune signature enriched for synaptic/neurotransmission and cytokine pathways, relevant to hepatic pathology and systemic immunity.

The neuroimmune signature identified in our study supports the growing body of evidence that immune cells utilize molecular mechanisms structurally and functionally analogous to neuronal synapses to coordinate complex immune responses during viral infections and neuroinflammation [17, 32, 33]. Although these so‐called “immune synapses” are not actual synaptic connections like those in the nervous system, they represent specialized, transient contact zones between immune cells that facilitate directed signaling and molecular exchange. In our analysis, genes encoding for components commonly associated with synaptic architecture, such as vesicle trafficking proteins, neurotransmitter receptors, adhesion molecules, and endocytic regulators, were consistently expressed and differentially regulated in both PBMCs and liver tissue across HBV, HCV, and HDV infections. These findings reinforce the concept that immune cells engage in sophisticated intercellular communication, and that such mechanisms may be co‐opted or disrupted during chronic inflammation and viral persistence [17, 18, 34].

Notably, our LDA highlighted the discriminatory power of neuroimmune genes to stratify infection in chronic conditions status on tissue compartments. Genes typically associated with synaptic development and plasticity, such as *NRG1*, *GRIN2B*, and *OLFM1* [35, 36, 37], emerged as central elements in distinguishing not only infected from noninfected individuals but also tissue‐specific transcriptional landscapes. These results support a reconceptualization of immune regulation as a hybrid communication network, in which immune cells use both canonical cytokine signaling and genes associated with synaptic pathways to also coordinate systemic responses [18, 32, 34].

The outcomes of our study reveal that, despite a clear divergence in transcriptional profiles that is both virus‐ and tissue‐specific, this heterogeneity is associated with distinct sets of neuroimmune genes. For instance, *GRIN2B* and *ADRA1B* are found in HBV‐infected liver, *ITGB1* and *IRF1* in HCV‐infected PBMCs, and *WDR62*, *EREG*, and *HP* in HDV‐infected liver. Functional enrichment analyses further support this divergence; while immune‐related pathways were broadly shared, nervous system processes such as axonogenesis and trans‐synaptic signaling were enriched primarily in HBV and HDV chronic liver infected samples. In contrast, neuropeptide signaling and neuron differentiation were more prominent in PBMCs from HBV and HCV infections. These patterns reflect underlying differences in viral biology, including chronicity and immune evasion strategies, which likely shape distinct neuroimmune landscapes [8, 37, 38]. Due to the limited availability of high‐quality datasets for HAV and HEV, their contributions remain less defined. However, they were included for comparative context and, in vitro, exhibited an absence of neuroimmune activity. Collectively, these findings underscore the need for virus‐specific analyses and highlight the importance of expanding available datasets to fully capture the diversity of neuroimmune responses across all hepatitis virus types.

The finding of active noradrenergic, IL‐1, and neuregulin ligand–receptor signaling axes, particularly in high‐grade HCC tumors, underscores the functional relevance of this neuroimmune machinery. Among these, the noradrenergic axis, anchored by the upregulated expression of *DBH* under the association of SLC6A2 and ADRA/ADRB receptors, emerged as the most dominant signaling pathway, especially in advanced disease. This mirrors findings from other solid tumors where adrenergic signaling promotes tumor growth, angiogenesis, and immune suppression [38, 39, 40]. These shared circuits between neurons and leukocytes suggest that the immune system is deeply embedded in the body's broader information‐processing network, modulated not only by inflammation but also by stress hormones and neurotransmitters [41].

Furthermore, genes linked to noradrenergic signaling, particularly those mediating β1‐adrenergic pathways, have been associated with immunosuppressive MAIT cell activity in HCC, suggesting that tumor immune escape may be driven by neuroimmune crosstalk [21, 37]. Our analysis supports this link between the expression of key noradrenergic‐related genes, such as DBH, SLC6A2, and their downstream ADRA/ADRB receptors, with increased tumor severity in TCGA‐LIHC samples. Network‐level visualization revealed that the noradrenaline, NRG1, and IL‐1 signaling axes form dominant ligand‐receptor frameworks in high‐grade HCC, with noradrenaline emerging as the most connected pathway. This reinforces the notion that neurotransmitter signaling actively participates in shaping the immune landscape of the tumor microenvironment. Additionally, neuroimmune alterations have been linked to liver pathology through stress‐induced glucocorticoid (GC) signaling, which impairs NK cell function and promotes immune evasion in liver cancer via the PD‐1/PD‐L1 pathway [42]. This neuroendocrine dysregulation, mediated by the HPA axis, may accelerate HCC progression and contribute to systemic symptoms observed in chronic hepatitis, reinforcing the neuroimmunome's potential as both a mechanistic link and a therapeutic target.

This highlights the therapeutic potential of targeting neuroimmune pathways. Indeed, strategies like vagal stimulation, β‐blockers, bioelectronic medicine, and celiac plexus neurolysis, already used in other contexts, could be repurposed to modulate sympathetic tone and reprogram immune responses in the tumor microenvironment [21, 26]. Integrating neuroimmune profiling into clinical practice may further help identify patients at higher risk and guide personalized therapies.

A particularly noteworthy observation was the collapse of neuroimmune gene co‐expression networks as HCC progressed. In early tumor stages (G1–G2), we observed robust, coherent correlations between genes involved in synaptic structure, cytokine regulation, and vesicle trafficking. This architecture disintegrated in late‐stage disease (G3–G4), where correlations were attenuated or even reversed, particularly among IL33, *OLFM1*, and *FER1L4*. This desynchronization may reflect a transition from immune surveillance and coordination to immune escape and systemic disintegration, a hallmark of malignant transformation [42].

Importantly, the presence of synapse‐associated genes in PBMCs, mirroring those found in hepatic tissue, underscores the systemic nature of the neuroimmunome [17, 21, 43]. Rather than acting in isolation, the liver and circulating immune cells appear to participate in a shared neuroimmune network that integrates metabolic, inflammatory, and neuronal signals across organs [21, 22, 32]. This network is likely modulated by afferent and efferent neural pathways, such as the vagus nerve and sympathetic innervation, reinforcing the concept that the immune system functions not merely as a defense mechanism but as a distributed sensing and communication system [18].

Our disease‐association analysis further supports this systems‐level view. Genes such as *DBH*, *WDR62*, *NRG1*, and *FER1L4*, identified in hepatic and blood immune transcriptomes, were also implicated in a broad range of disorders, including major depressive disorder, autoimmune diseases, and cancer, which have been previously associated with hepatitis [3, 10]. This convergence suggests that neuroimmune dysfunction may represent a transdiagnostic molecular pattern, indicating peripheral immune dysregulation linked to central nervous system comorbidities. These findings suggest that neuroimmune dysfunction may reflect a transdiagnostic mechanism linking peripheral immune dysregulation to CNS comorbidities [21]. Genes like *NRG1*, *OLFM1*, and *WDR62*, associated with both tumor progression and neuropsychiatric symptoms such as fatigue and depression, highlight the neuroimmune signature as a potential marker of disease severity and a target to mitigate extrahepatic manifestations in chronic hepatitis.

In conclusion, this study uncovers a coherent and dynamic neuroimmune signature that spans viral hepatitis, systemic immunity, and tumor progression. Our data support a paradigm in which the immune system is not merely a responder to infection and injury but also a participant in complex, multiorgan communication networks that include genes associated with synaptic features [17, 34, 43, 44]. Understanding this neuroimmune architecture opens new possibilities for therapeutic targeting, particularly of neurotransmitter‐modulated pathways such as the noradrenergic axis, and invites a reimagining of the liver as a neuroimmune interface in both health, disease, and the tumor microenvironment [36, 45].

Moreover, our findings open the door to exploring how neuroimmune disruption may interface with psychological stress and mood disorders in the context of hepatitis [9]. Previous studies have shown that patients with chronic hepatitis exhibit higher rates of psychological distress, including anxiety and depression, which may in turn influence disease progression through immunomodulatory pathways [10]. In this light, the neuroimmunome could serve as a mechanistic link between liver pathology and psychological states, supporting the integration of psychotherapeutic approaches as a complementary strategy in the clinical care of hepatitis patients.

## Author Contributions

Adriel Leal Nóbile, Anny Silva Adri, Júlia Nakanishi Usuda, Fernando Yuri Nery do Vale, Yohan Lucas Gonçalves Corrêa, Débora Gomes de Albuquerque Freitas, Roseane Galdioli Nava, Pedro Marçal Barcelos, Igor Salerno Filgueiras and Otávio Cabral‐Marques were responsible for conceptualization. Adriel Leal Nóbile was responsible for the analytic approach, performed statistical and R analysis, data curation, public data set collection and wrote the original manuscript draft, which was supervised by Otávio Cabral‐Marques. All authors contributed to critically revising and approved the final version of the manuscript for publication. The corresponding author (Adriel Leal Nóbile) attests that all listed authors meet authorship criteria and that no others meeting the criteria have been omitted.

## Conflicts of Interest

The authors declare no conflicts of interest.

## Supporting information

Reviewed Supporting Tables.

Reviewed Supporting Material.

Supp1.

Supp2.

Supp3.

Supp4.

Supp5.

Supp6.

Supp7.

Supp8.

supmat.

## Data Availability

The data that support the findings of this study are openly available in NCBI at https://www.ncbi.nlm.nih.gov/geo/query/acc.cgi, reference numbers GSE114916 (doi:10.1038/s41564‐019‐0425‐6), GSE13046 (doi:10.1128/JVI.02167‐08), GSE234478 (doi:10.1126/sciadv. adj4198), GSE118295 (doi:10.1128/JVI.00722‐18), GSE135860 (doi:10.1038/s41421‐021‐00337‐3), GSE126831 (doi:10.1053/j. gastro.2019.04.003), GSE211161 (doi:10.1016/j. jhepr.2022.100592), GSE29889 (doi:10.1371/journal. pone.0025584), GSE112118 (doi:10.1002/hep.30815), GSE135619 (doi:10.1073/pnas.1912307117), GSE224795 (doi:10.1016/j. antiviral.2023.105690), GSE53731 (doi:10.1002/jmv.24014), GSE107170 (doi:10.1158/1541‐7786. MCR‐18‐0012), GSE14668 (doi:10.1073/pnas.1003854107), GSE38941 (doi:10.1371/journal. pone.0049611), GSE47197 (doi:10.1371/journal. pone.0193232), GSE55092 (doi:10.1186/s12967‐014‐0230‐1), GSE65359 (doi:10.1093/infdis/jix683), GSE94660 (doi:10.1186/s12916‐017‐0973‐7), GSE164266 (doi:10.1136/gutjnl‐2020‐322182), GSE78737 (doi:10.1371/journal. ppat.1006916), GSE107170 (doi:10.1158/1541‐7786. MCR‐18‐0012), GSE154211 (doi:10.1111/liv.14772), GSE107170 (doi:10.1158/1541‐7786. MCR‐18‐0012), GSE98383 (doi:10.1158/1541‐7786. MCR‐18‐0012), GSE168048 (doi:10.3389/fmolb.2021.657631), GSE173897 (doi:10.1101/2025.08.19.25333999), GSE212871 (doi:10.1038/s41590‐021‐00982‐6), GSE65123 (doi:10.1002/jmv.24399), GSE93711 (doi:10.1016/j. immuni.2017.09.006), and TCGA database at https://portal.gdc.cancer.gov/projects/TCGA-LIHC. The data that supporting the findings of this study are available in the GEO repository at https://www.ncbi.nlm.nih.gov/geo/ and the TCGA portal at https://portal.gdc.cancer.gov/projects/TCGA-LIHC. These data were derived from the following publicly available resources: GEO accession numbers GSE114916, GSE13046, GSE234478, GSE118295, GSE135860, GSE126831, GSE211161, GSE29889, GSE112118, GSE135619, GSE224795, GSE53731, GSE107170, GSE14668, GSE38941, GSE47197, GSE55092, GSE65359, GSE78737, GSE94660, GSE154211, GSE98383, GSE168048, GSE173897, GSE212871, GSE65123, and GSE93711. The corresponding DOIs are listed in the Supporting Table 1a and suplementary material 1. All analysis scripts are publicly available on GitHub at https://github.com/adrielleal/neuroimmunome_hcc_severity.
