# Supplementary material for: The Neuroimmunome of Hepatitis Patients Associates With Disease Severity
Source: J Med Virol. 2025 Dec 5;97(12):e70742. doi: 10.1002/jmv.70742 (PMC12679811; doi:10.1002/jmv.70742)
Supplement: Supplementary file 2 — Reviewed Supporting Material. [file JMV-97-e70742-s006.docx]

**Supplemental Material**

**Data curation**

We analyzed transcriptomic data from the NCBI Gene Expression Omnibus (GEO) database, selecting human studies on hepatitis viruses (HAV–HEV) that utilized microarray or bulk RNA-seq platforms^1,2^. Inclusion criteria required negative controls and excluded confounding factors (e.g., alcohol use, autoimmune or chronic diseases), focusing on coding regions only **(Supplementarty Fig. 2)**. GEO datasets were grouped into: (a) *in vitro* (primary human hepatocytes [PHH], HepG2 hepatocellular carcinoma cells, and Huh7 human hepatoma cells)^3–5^, (b) PBMCs from acute and chronic hepatitis cases, and (c) liver tissue from chronically infected HCC patients. We included 27 datasets: GSE114916^6^, GSE13046^7^, GSE234478^8^, GSE118295^9^, GSE135860^10^, GSE126831^11^, GSE211161^12^, GSE29889^13^, GSE112118^14^, GSE135619^15^, GSE224795^16^, GSE53731^17^, GSE107170 (Has samples of HBV, HCV, and HDV)^18^, GSE14668^19^, GSE38941^20^, GSE47197^21^, GSE55092^22^, GSE65359^23^, GSE78737^24^, GSE94660^25^, GSE154211^26^, GSE98383^18^, GSE168048^27^, GSE173897^28^, GSE212871^29^, GSE65123^30^, and GSE93711^31^.

Each GSE dataset has its own method of viral diagnosis, patient clinical course, and timing of sample collection. However, not all of this information was always clearly provided. All available details and specific limitations for each GSE are described in Supplementary Table 1a.

We analyzed 1,387 samples: 959 liver (474 controls, 485 infected), 192 PBMC (92 controls, 100 infected), and 236 in vitro samples (113 controls, 123 infected). Based on our inclusion criteria, HAV and HEV were studied exclusively in vitro, HBV and HCV across all tissue types, and HDV only in liver. Diagnostic methods included RT-PCR, viral load quantification, and serological assays (e.g., anti-HBc, anti-HCV). Clinical courses ranged from acute cases (including acute liver failure) to chronic infections with histological confirmation of HCC. Gene expression analyses were typically performed by comparing infected and non-infected samples or stratifying by disease progression and viral load. See Supplementary Table 1a for diagnostic methods, sample details, and check the original GSE references.

**Differential expression analysis**

Differential expression analysis was performed using DESeq2 for bulk RNA-seq data and Limma for microarray datasets. Genes were considered significantly differentially expressed if they exhibited a log2 fold change (Log2FC) greater than 1 or less than -1, along with an adjusted p-value below 0.05 ^32–34^. Comparisons were made between infected and control samples across *in vitro* (tumor vs. non-tumor cells), PBMC (serologically infected vs. non-infected), liver tissues (tumor vs. non-tumor, histologically confirmed), and The Cancer Genome Atlas Program (TCGA) HCC samples (grades G2–G4 vs. G1 as reference).

To account for potential confounders, RNA-seq analyses using DESeq2 incorporated diagnostic groupings (e.g., infected vs. control, HCC vs. non-tumoral, seropositive vs. seronegative) as primary design factors. Due to incomplete annotation, covariates such as age and sex were not consistently available across datasets and were therefore excluded^35^. For Limma-based analyses, batch effects, particularly in patient-derived datasets, were corrected using the Empirical Bayes (EB) method implemented in ComBat (non-parametric prior)^36^. In both approaches, samples exposed to pharmacological treatments were excluded to avoid confounding^37^. No substantial sources of variation beyond disease status were detected, supporting the biological specificity of the observed transcriptional alterations.

**Meta-analysis**

A comprehensive gene meta-analysis was conducted using the MetaVolcanoR package, applying the 'Combining MetaVolcano' approach to differential expression results from datasets, which combines adjusted p-value and log2 fold change (Log2FC) to compute consensus DEGs^38^. To capture virus- and tissue-specific responses, we performed ten separate meta-analyses across experimental contexts: five *in vitro* (HAV, HBV, HCV, HDV, HEV), three in liver tissue (HBV, HCV, HDV), and two in PBMCs (HBV, HCV), based on the availability of sufficient datasets to support robust integrative analysis. Standard parameters were used to ensure consistency, with an adjusted p-value < 0.05, and thresholds of Log2FC < -1 (downregulated) and Log2FC > 1 (upregulated). Gene-level statistics were aggregated using Fisher’s method for adjusted p-value combination across studies. Identified metaDEGs were further classified by gene type (e.g., protein-coding, miRNA, snoRNA, lncRNA, non-coding) using the ShinyGO platform, providing insights into the genomic features underlying the observed expression changes (Supplementary Fig. 3b)^39,40^. For viral hepatitis types represented by multiple datasets across acute and chronic phases, such as HBV and HCV, we computed the mean dysregulation (log2FC) of shared genes across studies, allowing us to generate a unified signature that reflects overall immune activity, irrespective of clinical phase. This decision was supported by our earlier findings showing a similar DEGs enriched signature linked to immune and nervous system BP in both acute and chronic contexts (**Supplementary Fig. 3c**), suggesting that combining these data preserves relevant functional signals. ^39,40^

**Enrichment, Clustering, and Interactome Analyses**

Gene ontology (GO)^41^ enrichment analysis was performed using EnrichR^42^ (adj. p < 0.05) to identify neuroimmune-related biological processes (BPs), based on nervous (e.g., "Synapse", "Neurotransmitter") and immune (e.g., "Cytokine", "Leukocyte") keyword filters (**Supplementary Table 1f**). We used DEGs from acute and chronic GSEs to compare signatures across different time points, and metaDEGs to define a general neuroimmune signature of viral hepatitis. Gene and BP associations were further explored using the Appyters platform, where term frequency-inverse document frequency (TF-IDF) values were computed for each GO term’s gene set, and values were embedded using UMAP for dimensionality reduction. Leiden clustering was applied to identify functionally related BP clusters^43^. Network analysis of metaDEGs and enriched BPs was conducted using ggnet2 in R to map neuroimmune interactomes^44^. Additionally, metaDEGs associated with synaptic functions were characterized using the SynGO (2024) tool via EnrichR, enabling functional annotation and exploration of their roles and interactions in synaptic biology within the study context^45^.

**Linear discriminant analysis of nervous and immune BP-related genes**

We applied Linear Discriminant Analysis (LDA) on GEO datasets with the largest sample sizes, focusing on the conditions that revealed nervous and immune gene interactions, GSE94660 and GSE164266 via SVA integration (HBV/liver), namely GSE173897 (HBV/PBMC), GSE154211 (HCV/liver), GSE212871 (HCV/PBMC), and GSE107170 (HDV/liver), to identify neuroimmune genes that best discriminate between control and infected groups (**Supplementary Table 3a**). LDA models were trained using 70% of the samples and tested on the remaining 30%, with classification performance evaluated by accuracy. Models with accuracy below 50% were considered ineffective. Genes with the highest LDA coefficients were interpreted as positively contributing to the separation of infected samples from controls, while those with strongly negative coefficients contributed to the control group discrimination^46^.

**Using TCGA database to evaluate LDA-selected genes in cancer severity**

Since no publicly available GEO datasets included HCC grade annotations necessary to assess the relationship between gene expression and tumor grade, we turned to The Cancer Genome Atlas Liver Hepatocellular Carcinoma (TCGA-LIHC) dataset for further analysis^2^. A total of 430 samples were retrieved, stratified into four histological grades: G1 (n = 62, “control”), G2 (n = 213), G3 (n = 142), and G4 (n = 13) (**Supplementary Fig. 7a**). Unlike the virus-stratified datasets, the TCGA cohort included a limited number of viral hepatitis cases: HBV-infected samples had only seven in G1 and one in G4, while HCV-infected samples lacked G4 cases entirely and had just five in G1 and two in G3. In contrast, non-viral HCC samples were the most prevalent across all grades (G1 = 31, G2 = 115, G3 = 84, G4 = 12). Due to this imbalance, a severity analysis specific to viral hepatitis–associated HCC was not feasible (**Supplementary Fig. 7b**). Therefore, we removed the batch effect related to viral etiology and focused exclusively on gene expression changes associated with HCC severity.

The transcriptomic data were accessed through the UCSCXenaTools package (v.41)^47^. To address potential confounding variables, DESeq2’s model design incorporated experimental groupings (G1 vs. G2, G3, G4) as primary factors. Covariates such as age, sex, and histopathological carcinoma classification were included in the design formula to correct for potential batch effects; however, variables with incomplete annotation across datasets were excluded from the final model (**Supplementary Table 1a**).

Genes identified by LDA as stratifiers for the infected group were intersected with DEGs from TCGA^2^ (each grade vs. G1) using UpSet plots and Venn diagrams. Pairwise gene expression comparisons between HCC histological grades (e.g., G1 vs. G2, G1 vs. G3, G1 vs G4) were performed using the Wilcoxon rank-sum test, with false discovery rate (FDR) correction (adjusted p < 0.05), and genes were enriched for BP and synapse-related pathways using EnrichR and SynGO^42,45^. MANOVA with Bootstrap resampling (using 1000 iterations) evaluated gene expression differences across grades, ensuring robustness in transcriptomic data^48^. Spearman's correlation assessed the relationship between gene expression and HCC grades^49^. These genes were compared based on their log2FC and adjusted p-values (FDR < 0.05), as derived from both TCGA datasets and a viral hepatitis meta-analysis.

**References:**

1. Clough, E. *et al.* NCBI GEO: archive for gene expression and epigenomics data sets: 23-year update. *Nucleic Acids Res* **52**, D138–D144 (2024).

2. Wang, Z., Jensen, M. A. & Zenklusen, J. C. A Practical Guide to The Cancer Genome Atlas (TCGA). *Methods in Molecular Biology* **1418**, 111–141 (2016).

3. Zeilinger, K., Freyer, N., Damm, G., Seehofer, D. & Knöspel, F. Cell sources for in vitro human liver cell culture models. *Exp Biol Med* **241**, 1684–1698 (2016).

4. Pinti, M. *et al.* Hepatoma HepG2 cells as a model for in vitro studies on mitochondrial toxicity of antiviral drugs: which correlation with the patient? *J Biol Regul Homeost Agents* **17**, 166–171 (2003).

5. Fouillé, R. *et al.* A novel in vitro system for simultaneous infections with hepatitis B, C, D and E viruses. *JHEP Reports* **7**, 101383 (2025).

6. Yamane, D. *et al.* Basal expression of interferon regulatory factor 1 drives intrinsic hepatocyte resistance to multiple RNA viruses. *Nat Microbiol* **4**, 1096–1104 (2019).

7. Larrea, E. *et al.* Oncostatin M enhances the antiviral effects of type I interferon and activates immunostimulatory functions in liver epithelial cells. *J Virol* **83**, 3298–3311 (2009).

8. Shiota, T. *et al.* Hepatoviruses promote very-long-chain fatty acid and sphingolipid synthesis for viral RNA replication and quasi-enveloped virus release. *Sci Adv* **9**, (2023).

9. Xia, Y. *et al.* Hepatitis B Virus Deregulates the Cell Cycle To Promote Viral Replication and a Premalignant Phenotype. *J Virol* **92**, (2018).

10. Yuan, S. *et al.* Multiomics interrogation into HBV (Hepatitis B virus)-host interaction reveals novel coding potential in human genome, and identifies canonical and non-canonical proteins as host restriction factors against HBV. *Cell Discov* **7**, (2021).

11. Lupberger, J. *et al.* Combined Analysis of Metabolomes, Proteomes, and Transcriptomes of Hepatitis C Virus-Infected Cells and Liver to Identify Pathways Associated With Disease Development. *Gastroenterology* **157**, 537-551.e9 (2019).

12. Edwards, J. S. *et al.* Downregulation of SOCS1 increases interferon-induced ISGylation during differentiation of induced-pluripotent stem cells to hepatocytes. *JHEP Reports* **4**, (2022).

13. MacPherson, J. I. *et al.* An integrated transcriptomic and meta-analysis of hepatoma cells reveals factors that influence susceptibility to HCV infection. *PLoS One* **6**, (2011).

14. Winer, B. Y. *et al.* Analysis of Host Responses to Hepatitis B and Delta Viral Infections in a Micro-scalable Hepatic Co-culture System. *Hepatology* **71**, 14–30 (2020).

15. Todt, D. *et al.* Robust hepatitis E virus infection and transcriptional response in human hepatocytes. *Proc Natl Acad Sci U S A* **117**, 1731–1741 (2020).

16. Kinast, V. *et al.* Janus kinase-inhibition modulates hepatitis E virus infection. *Antiviral Res* **217**, (2023).

17. Zhang, F. *et al.* Hepatitis E genotype 4 virus from feces of monkeys infected experimentally can be cultured in PLC/PRF/5 cells and upregulate host interferon-inducible genes. *J Med Virol* **86**, 1736–1744 (2014).

18. Diaz, G. *et al.* Molecular Signature and Mechanisms of Hepatitis D Virus-Associated Hepatocellular Carcinoma. *Mol Cancer Res* **16**, 1406–1419 (2018).

19. Farci, P. *et al.* B cell gene signature with massive intrahepatic production of antibodies to hepatitis B core antigen in hepatitis B virus-associated acute liver failure. *Proc Natl Acad Sci U S A* **107**, 8766–8771 (2010).

20. Nissim, O. *et al.* Liver regeneration signature in hepatitis B virus (HBV)-associated acute liver failure identified by gene expression profiling. *PLoS One* **7**, (2012).

21. Halgand, B. *et al.* Hepatitis B Virus Pregenomic RNA in Hepatocellular Carcinoma: A Nosological and Prognostic Determinant. *Hepatology* **67**, 86–96 (2018).

22. Melis, M. *et al.* Viral expression and molecular profiling in liver tissue versus microdissected hepatocytes in hepatitis B virus-associated hepatocellular carcinoma. *J Transl Med* **12**, (2014).

23. Liu, H. *et al.* Differentially Expressed Intrahepatic Genes Contribute to Control of Hepatitis B Virus Replication in the Inactive Carrier Phase. *J Infect Dis* **217**, 1044–1054 (2018).

24. Sekhar, V. *et al.* Infection with hepatitis C virus depends on TACSTD2, a regulator of claudin-1 and occludin highly downregulated in hepatocellular carcinoma. *PLoS Pathog* **14**, (2018).

25. Yoo, S. *et al.* A pilot systematic genomic comparison of recurrence risks of hepatitis B virus-associated hepatocellular carcinoma with low- and high-degree liver fibrosis. *BMC Med* **15**, 214 (2017).

26. Wang, S. C. *et al.* Exosome-derived differentiation antagonizing non-protein coding RNA with risk of hepatitis C virus-related hepatocellular carcinoma recurrence. *Liver Int* **41**, 956–968 (2021).

27. Ma, S. *et al.* Identification of a Potential miRNA-mRNA Regulatory Network Associated With the Prognosis of HBV-ACLF. *Front Mol Biosci* **8**, (2021).

28. Silvey, S., Olex, A., Tang, S. & Liu, J. Sample Size Requirements for Machine Learning Classification of Binary Outcomes in Bulk RNA-Seq Data. *medRxiv* 2025.08.19.25333999 (2025) doi:10.1101/2025.08.19.25333999.

29. Tonnerre, P. *et al.* Differentiation of exhausted CD8+ T cells after termination of chronic antigen stimulation stops short of achieving functional T cell memory. *Nat Immunol* **22**, 1030–1041 (2021).

30. Zabaleta, A. *et al.* Gene expression analysis during acute hepatitis C virus infection associates dendritic cell activation with viral clearance. *J Med Virol* **88**, 843–851 (2016).

31. Wolski, D. *et al.* Early Transcriptional Divergence Marks Virus-Specific Primary Human CD8+ T Cells in Chronic versus Acute Infection. *Immunity* **47**, 648-663.e8 (2017).

32. Love, M. I., Huber, W. & Anders, S. Moderated estimation of fold change and dispersion for RNA-seq data with DESeq2. *Genome Biol* **15**, (2014).

33. Sanchis, P. *et al.* Analysis workflow of publicly available RNA-sequencing datasets. *STAR Protoc* **2**, 100478 (2021).

34. Ritchie, M. E. *et al.* limma powers differential expression analyses for RNA-sequencing and microarray studies. *Nucleic Acids Res* **43**, e47–e47 (2015).

35. Roy, J., Monthony, A. S. & Torkamaneh, D. DESeq2-MultiBatch: Batch Correction for Multi-Factorial RNA-seq Experiments. *bioRxiv* 2025.04.20.649392 (2025) doi:10.1101/2025.04.20.649392.

36. Kupfer, P. *et al.* Batch correction of microarray data substantially improves the identification of genes differentially expressed in Rheumatoid Arthritis and Osteoarthritis. *BMC Med Genomics* **5**, 1–12 (2012).

37. Zhou, W., Koudijs, K. K. M. & Böhringer, S. Influence of batch effect correction methods on drug induced differential gene expression profiles. *BMC Bioinformatics* **20**, 1–14 (2019).

38. MetaVolcanoR: Differential expression meta-analysis tool. https://bioconductor.statistik.tu-dortmund.de/packages/3.18/bioc/vignettes/MetaVolcanoR/inst/doc/MetaVolcano.html.

39. Ge, S. X., Jung, D., Jung, D. & Yao, R. ShinyGO: a graphical gene-set enrichment tool for animals and plants. *Bioinformatics* **36**, 2628–2629 (2020).

40. Huo, Z., Tang, S., Park, Y. & Tseng, G. P-value evaluation, variability index and biomarker categorization for adaptively weighted Fisher’s meta-analysis method in omics applications. *Bioinformatics* **36**, 524–532 (2020).

41. Ashburner, M. *et al.* Gene Ontology: tool for the unification of biology. *Nature Genetics 2000 25:1* **25**, 25–29 (2000).

42. Xie, Z. *et al.* Gene Set Knowledge Discovery with Enrichr. *Curr Protoc* **1**, e90 (2021).

43. Clarke, D. J. *et al.* Appyters: Turning Jupyter Notebooks into data-driven web apps. *Patterns* **2**, 100213 (2021).

44. ggnet2: network visualization with ggplot2. https://briatte.github.io/ggnet/.

45. Koopmans, F. *et al.* SynGO: An Evidence-Based, Expert-Curated Knowledge Base for the Synapse. *Neuron* **103**, 217-234.e4 (2019).

46. Zhao, S., Zhang, B., Yang, J., Zhou, J. & Xu, Y. Linear discriminant analysis. *Nature Reviews Methods Primers 2024 4:1* **4**, 1–16 (2024).

47. Wang, S. & Liu, X. The UCSCXenaTools R package: a toolkit for accessing genomics data from UCSC Xena platform, from cancer multi-omics to single-cell RNA-seq. *J Open Source Softw* **4**, 1627 (2019).

48. Konietschke, F., Bathke, A. C., Harrar, S. W. & Pauly, M. Parametric and nonparametric bootstrap methods for general MANOVA. *J Multivar Anal* **140**, 291–301 (2015).

49. Wissler, C. The Spearman correlation formula. *Science (1979)* **22**, 309–311 (1905).
