# Supplementary material for: The Neuroimmunome of Hepatitis Patients Associates With Disease Severity
Source: J Med Virol. 2025 Dec 5;97(12):e70742. doi: 10.1002/jmv.70742 (PMC12679811; doi:10.1002/jmv.70742)
Supplement: Supplementary file 3 — Supp1. [file JMV-97-e70742-s001.pdf]

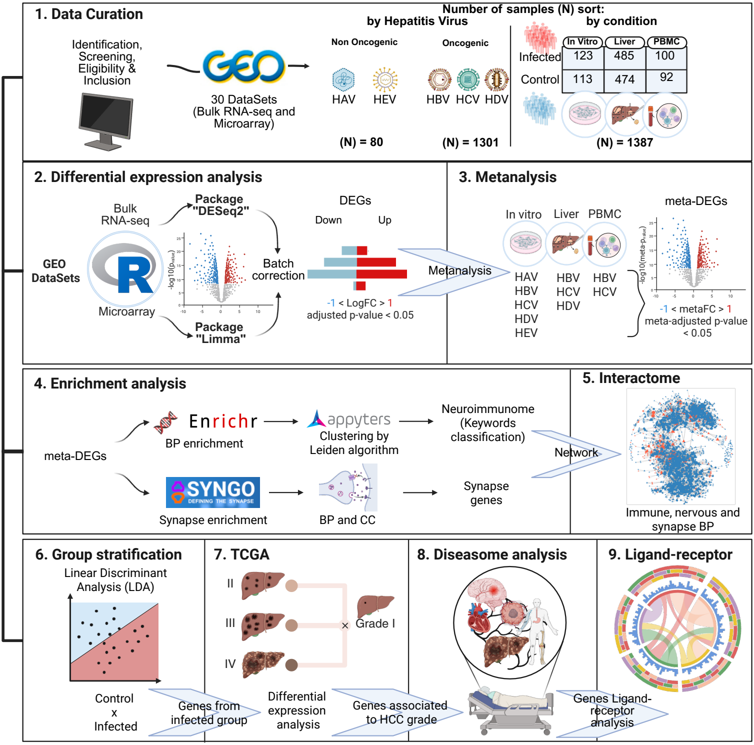
1. Data Curation
Identification, Screening, Eligibility & Inclusion
30 DataSets (Bulk RNA-seq and Microarray)
Number of samples (N) sort:
by Hepatitis Virus
Non Oncogenic
Oncogenic
HAV
HEV
HBV
HCV
HDV
(N) = 80
(N) = 1301
by condition
In Vitro
Liver
PBMC
Infected
123
485
100
Control
113
474
92
(N) = 1387
2. Differential expression analysis
Bulk RNA-seq
GEO DataSets
Microarray
Package "DESeq2"
Package "Limma"
Batch correction
-log10(p_value)
DEGs
Down
Up
-1 < LogFC > 1
adjusted p-value < 0.05
Metanalysis
3. Metanalysis
In vitro
Liver
PBMC
HAV
HBV
HCV
HDV
HEV
HBV
HCV
HDV
HBV
HCV
meta-DEGs
-log10(meta-p_value)
-1 < metaFC > 1
meta-adjusted p-value < 0.05
4. Enrichment analysis
meta-DEGs
Enrichr
BP enrichment
appyters
Clustering by Leiden algorithm
Neuroimmunome (Keywords classification)
SYNGO
DEFINING THE SYNAPSE
Synapse enrichment
BP and CC
Synapse genes
Network
5. Interactome
Immune, nervous and synapse BP
6. Group stratification
Linear Discriminant Analysis (LDA)
Control x Infected
Genes from infected group
7. TCGA
II
III
IV
Grade I
Differential expression analysis
Genes associated to HCC grade
8. Diseasome analysis
9. Ligand-receptor
Genes Ligand-receptor analysis
