# Supplementary figures and images for: The Neuroimmunome of Hepatitis Patients Associates With Disease Severity
Source: J Med Virol. 2025 Dec 5;97(12):e70742. doi: 10.1002/jmv.70742 (PMC12679811; doi:10.1002/jmv.70742)

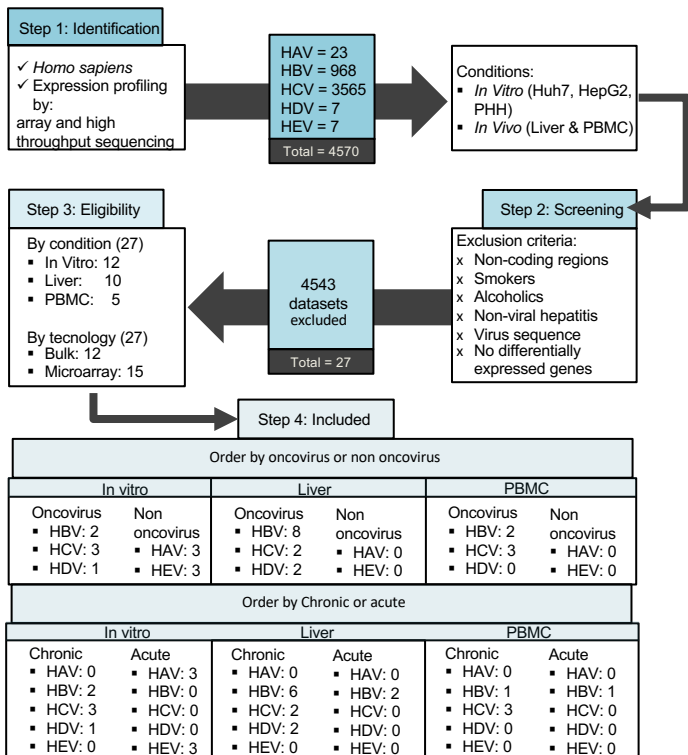

Supplement: Supplementary file 4 — Supp2. [file JMV-97-e70742-s010.pdf]

a

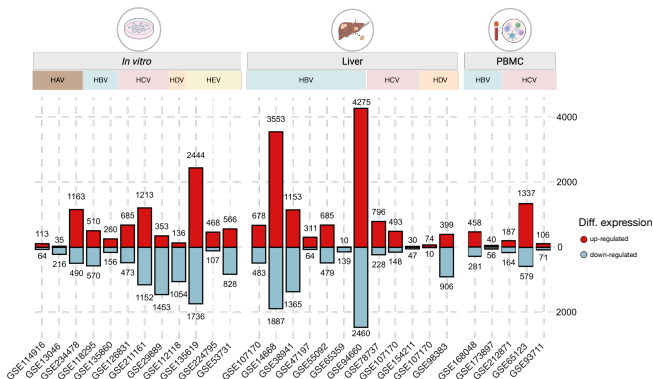

b

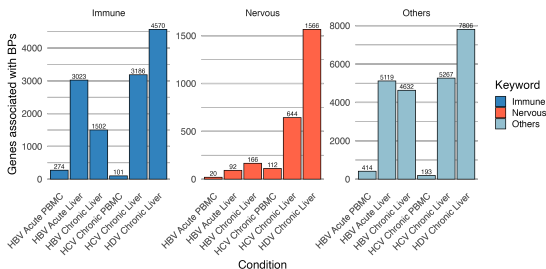

c

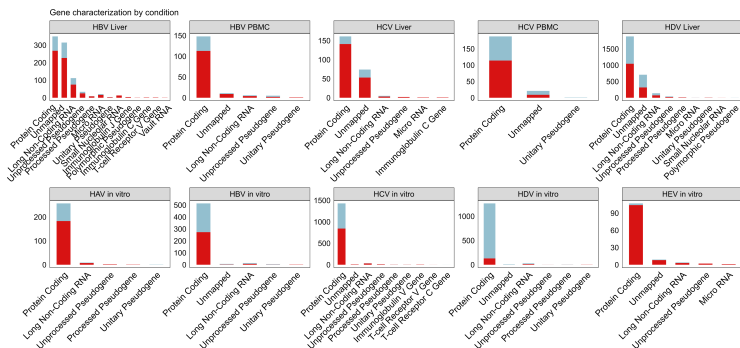

Supplement: Supplementary file 5 — Supp3. [file JMV-97-e70742-s004.pdf]

**a**

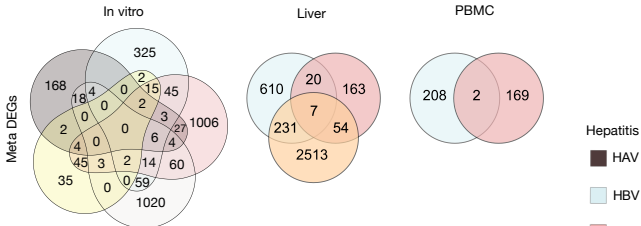

b

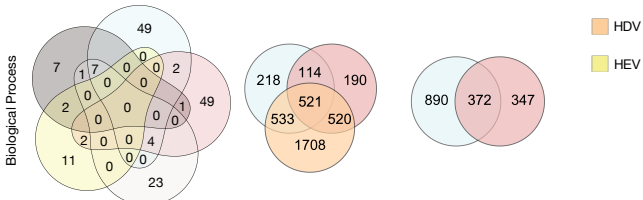

1

Supplement: Supplementary file 6 — Supp4. [file JMV-97-e70742-s009.pdf]

a

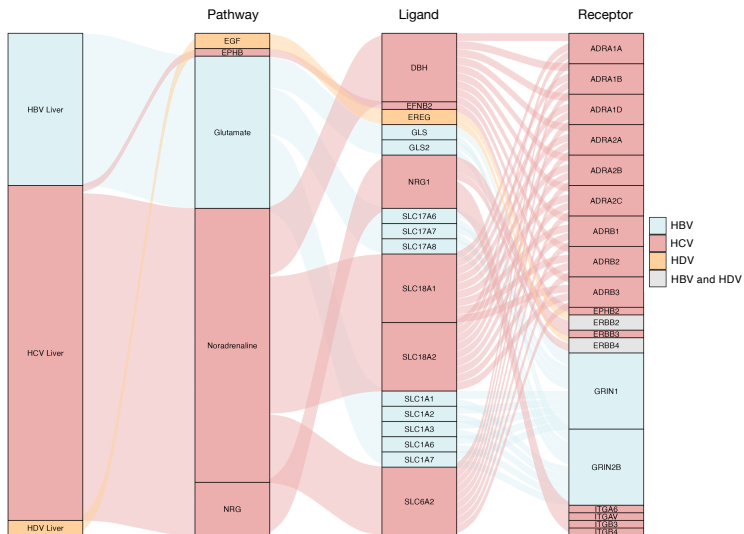

Supplement: Supplementary file 7 — Supp5. [file JMV-97-e70742-s007.pdf]

**a**

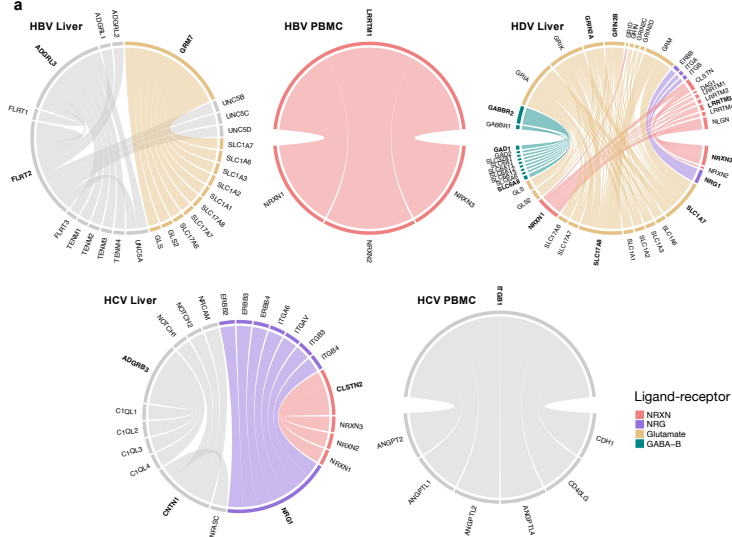

**b**

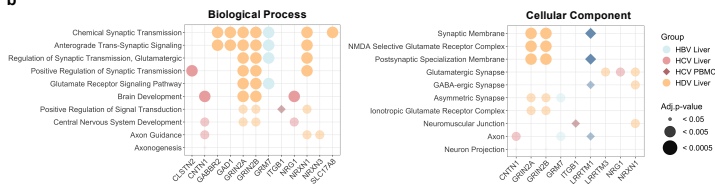

Supplement: Supplementary file 8 — Supp6. [file JMV-97-e70742-s003.pdf]

**a**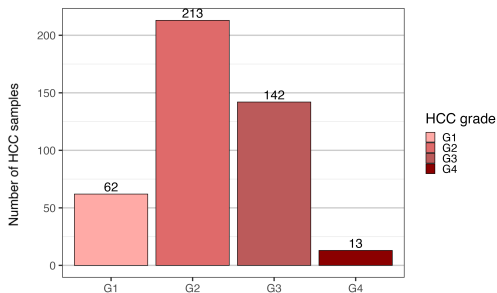**b**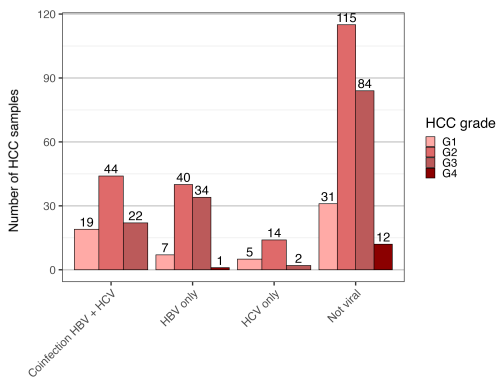

Supplement: Supplementary file 9 — Supp7. [file JMV-97-e70742-s011.pdf]

**a**

TCGA – Wilcoxon rank-sum with FDR correction

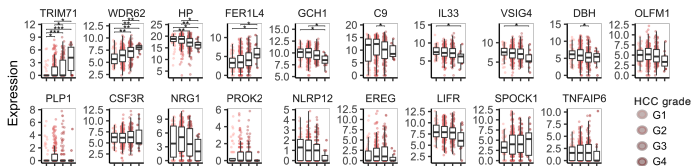

**b**

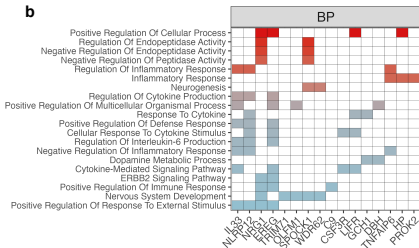

**c**

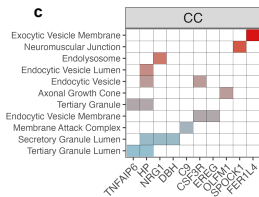

**d**

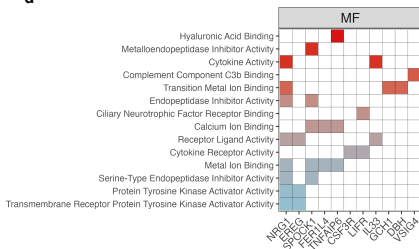

**e**

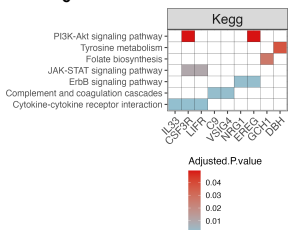

Supplement: Supplementary file 10 — Supp8. [file JMV-97-e70742-s002.pdf]
