# Supplementary material for: The Neuroimmunome of Hepatitis Patients Associates With Disease Severity
Source: J Med Virol. 2025 Dec 5;97(12):e70742. doi: 10.1002/jmv.70742 (PMC12679811; doi:10.1002/jmv.70742)
Supplement: Supplementary file 11 — supmat. [file JMV-97-e70742-s008.docx]

**Supplementary Figure legends**

**Supplementary 1 –** **Step by step of the methods employed.** This workflow outlines the analytical steps from data curation to differential expression, meta-analysis, functional enrichment, interactome construction, and integration with clinical and molecular data.

**Supplementary 2 – Flowchart of dataset inclusion**. From an initial pool of 4,570 hepatitis virus-related transcriptomic datasets identified in GEO, 27 were retained following quality control and eligibility screening (e.g., control availability, absence of co-infections, design compatibility). These comprised 12 in vitro (HAV ▒=▒ 3, HEV ▒=▒ 3, HBV ▒=▒ 2, HCV ▒=▒ 3, HDV ▒=▒ 1), 10 liver (HBV ▒=▒ 8, HCV ▒=▒ 2, HDV ▒=▒ 2), and 5 PBMC datasets (HBV ▒=▒ 2, HCV ▒=▒ 3). No liver or PBMC datasets were retained for HAV or HEV. A total of 4,543 datasets were excluded.

**Supplementary 3 –** **Differential Gene Expression and Functional Characterization Across Conditions.** (a) A bar plot shows the number of significantly upregulated (red) and downregulated (blue) genes across 27 transcriptomic datasets, stratified by virus type (HAV, HBV, HCV, HDV, HEV). (b) DEGs from differential expression analysis of acute HBV liver infection (GSE14668), chronic HBV liver infection (GSE94660 and GSE164266), acute HBV PBMC infection (GSE168048), chronic HCV liver infection (GSE78737), chronic HCV PBMC infection (GSE65123), and chronic HDV liver infection (GSE98383) were associated with the nervous, immune, and other systems, based on keyword classification. (c) Functional categorization of meta-differentially expressed genes across all conditions. Most DEGs were classified as protein-coding genes, with a notable presence of non-coding RNAs (e.g., lncRNAs, pseudogenes).

**Supplementary 4 – Overlap and functional enrichment of meta-DEGs across hepatitis viruses and tissue compartments.** (a) Venn diagrams illustrate the intersection of significantly meta-differentially expressed genes (meta-DEGs) across in vitro (left), liver (middle), and PBMC (right) conditions for hepatitis A-E viruses. While virus-specific responses were observed, only a small subset of genes was shared across infections: 0 in vitro, 7 in liver, and 2 in PBMC datasets. (b) Venn diagrams show the overlap of enriched biological processes (BPs) among immune- and nervous-related gene sets, stratified by condition (in vitro, liver, PBMC). Each virus–tissue combination exhibited varying degrees of neuroimmune convergence, with the greatest overlap observed in liver and none in in vitro datasets.

**Supplementary 5 – Ligand-receptor analysis, LDA genes**. A Sankey diagram illustrates neurotransmitter-related ligand-receptor interactions inferred from LDA segregated into HBV (blue), HCV (red), and HDV-infected (orange) genes within liver tissues. Noradrenergic signaling (e.g., DBH, SLC18A1/2, SLC6A2) emerged as a shared axis across viruses, while glutamatergic transporters (SLC17A6-8) and receptors (GRIN1, GRIN2B) were linked to HBV and HCV. HDV uniquely engaged EGF-EPHB signaling, and neuregulin-mediated ERBB interactions were restricted to HCV and HDV. Ligands and transporters are shown in the center-right, converging on receptors (far right) from the ADRA, ADRB, ERBB, and GRIN families. This suggests virus-specific yet intersecting neurotransmitter communication modules in the hepatic microenvironment, beyond immune-related pathways.

**Supplementary 6 – Functional landscape of synapse-related ligand-receptor genes across hepatitis virus infections.** (a) Chord diagrams depict ligand-receptor interactions among synapse-associated meta-DEGs across HBV, HCV, and HDV in liver and PBMC samples. Arcs are color-coded by neurotransmission-related pathways: glutamatergic (purple), noradrenergic (red), neuregulin (orange), and GABA-B (teal). Gray arcs represent ligand–receptor connections not linked to classical neurotransmission, providing context for non-synaptic signaling. Genes identified in the meta-analysis and annotated to synaptic ontologies are shown in bold. The center-right area of each diagram displays the corresponding ligands. The variable presence of neurotransmitter pathways across virus–tissue combinations highlights potential virus-specific modulation of neural-like signaling in hepatic immune niches. (b) Functional enrichment of synapse-linked ligand-receptor genes. Left panel panel shows Gene Ontology (GO) Biological Processes (BP), showing enrichment in synaptic transmission, glutamate signaling, axon guidance, and brain development. The right panel indicates GO Cellular Components (CC), showing localization to synaptic membranes, NMDA receptor complexes, and neuromuscular junctions. Dot size represents significance (adjusted p-value), and color indicates the associated virus–tissue condition. These findings support the neurofunctional relevance of the identified ligand-receptor interactions in hepatitis-associated immune regulation.

**Supplementary 7 – Distribution of HCC histological grades in TCGA-LIHC cohort.** (a) The overall distribution of hepatocellular carcinoma (HCC) cases across histological grades (G1 to G4) in the TCGA dataset (n▒=▒430). (b) Stratification of HCC grade by viral status, including co-infection with HBV▒+▒HCV, HBV only, HCV only, and non-viral cases. Viral annotation was derived from TCGA metadata. The majority of G3 and G4 tumors occurred in non-viral and HBV-infected patients, whereas lower-grade tumors (G1–G2) were more evenly distributed. Counts represent the number of patients per group.

**Supplementary 8 – Expression profiles and functional enrichment of neuroimmune genes associated with HCC severity.** (a) Boxplots displaying normalized expression levels of 19 neuroimmune genes identified as differentially expressed across hepatocellular carcinoma (HCC) histological grades. Genes such as TRIM71, WDR62, DBH, FER1L4, and IL33 show progressive upregulation from grade G1 to G4. (b–e) Enrichment analysis of the 19 genes using Gene Ontology and KEGG databases: (b) GO: Biological Processes (BP), highlighting roles in immune response regulation, cytokine signaling, dopaminergic signaling, and neurodevelopmental processes. (c) GO: Cellular Components (CC), revealing associations with exocytic vesicles, neuromuscular junctions, and axonal structures. (d) GO: Molecular Functions (MF), showing enrichment in receptor and cytokine activity, metallopeptidase inhibition, and neurotrophic factor binding. (e) KEGG pathway enrichment, including PI3K-Akt, JAK–STAT, and ERBB signaling pathways, supporting the involvement of these genes in oncogenic and immunoregulatory networks. Color intensity in all heatmaps reflects adjusted p-values (FDR), with darker shades indicating stronger statistical significance.
